# Kinetic Resolution: A Powerful Tool for the Synthesis of Planar-Chiral Ferrocenes

**DOI:** 10.3390/molecules14114747

**Published:** 2009-11-20

**Authors:** Andrea-Nekane R. Alba, Ramon Rios

**Affiliations:** Department of Organic Chemistry, University of Barcelona, Martí I Franqués 1-11 08028, Barcelona, Spain

**Keywords:** ferrocene, kinetic resolution, planar chirality

## Abstract

Since the serendipitous discovery of ferrocene by Pauson and Kealy in 1951, it has become one of the most important structures in Organic Chemistry. Lately, kinetic resolution has emerged as a useful tool for the synthesis of planar chiral ferrocenes. This review aims to cover and discuss the development of this topic.

## 1. Introduction

Chiral ferrocenes are of interest in several areas. Their main use is in asymmetric catalysis as chiral ligands of transition-metal complexes [1]. Applications have been found in asymmetric hydrogenation [2], hydrosilylation [3,4], aldolization [5,6], Michael additions [7], cross coupling reactions [8], and asymmetric additions of diethyl zinc to aldehydes [9].

Chiral ferrocenes have also been used as modular units in materials science, e.g. in ferroelectric liquid crystals or in non-linear optics [10]. Finally, chiral ferrocenes could be of importance as structural unit of products with biological or biochemical activities [11]. In this review, we intend to present the various ways to prepare enantiopure chiral planar ferrocenes *via* kinetic resolution.

Enantiopure 1,2-disubstituted ferrocenes with both planar and central chirality can be obtained with the procedure described by Ugi *et al*.: diastereoselective *ortho*-lithiation with *n*-BuLi of homochiral (α-dimethylamino)ethylferrocene followed by reaction with a suitable electrophile [12]. In addition to this method, a chiral acetal of ferrocenecarboxaldehyde can be used as substrate for *ortho*-lithiation [13]. Electrophilic quenching of the metallated compound and subsequent removal of the chiral moiety affords enantiopure *ortho*-substituted ferrocenecarboxaldehydes with planar chirality. Other *ortho*-directing auxiliary groups which allow metallation of enantiopure ferrocenes in order to attain planar chirality are oxazolines [14,15,16] or sulfoxides [17]. Snieckus’ enantioselective *ortho*-metallation of achiral ferrocene carboxamides by the butyllithium–sparteine system also gives access to planar-chiral ferrocenes [18].

Enzymatic resolution of 1,2-disubstituted ferrocenes is, on the other hand, a different approach for the preparation of enantiopure 1,2-disubstituted ferrocenyl compounds to be used as starting materials in the synthesis of different types of enantiopure ferrocenes of established planar chirality. These procedures avoid the need of stoichiometric sources of chirality. Furthermore, as it is still much easier and less expensive to access racemates, so resolution strategies must always be carefully evaluated against any asymmetric process [19].

## 2. Enzymatic Kinetic Resolutions

**Scheme 1:**
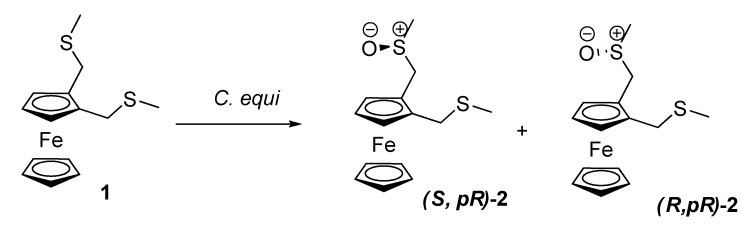
Desymmetrization of 1,2-bis(methylthiomethyl)ferrocene (**1**)

In 1990, Yamazaki *et al*. oxidized 1,2-bis(methylthiomethyl)ferrocene (**1**) with *Corynebacterium equi* IFO 3730 to give monosulfoxide **2** (see Scheme 1) in two diastereomeric forms (4:1) [20]. This constituted a desymmetrization of a prochiral ferrocene. Two diastereomers which shared the same planar chirality were obtained as a result; that is, the bacterial monooxygenase generated specific planar chirality, even if it was not very stereoselective in forming a chiral center about the sulphur atom. Anyway, its enantiotopic differentiation between two side chains was considerable.

Until early the 90s, most of the reported studies using enzymes to effect selective transformations were concerned with central chirality. In 1992, Izumi and co-workers reported the enzymatic kinetic resolution of [4](1,2)ferrocenophane derivatives [21].

In order to perform the resolution of (±)-[4](1,2)ferrocenophan-1-one (**3**), they had to subject enol acetate derivative **4** to hydrolysis by commercial lipases, because bakers’ yeast did not effect the reduction of **3**. When enol acetate **4** was incubated with the lipase of *Pseudomonas fluorescens* (**7**) in phosphate buffer, the hydrolysis proceeded rapidly to afford optically active **(+)-(*R*)-3**, with 24% ee, and recovered enol acetate **(+)-(*S*)-4** with very high enantiomeric excess (> 99%).

**Scheme 2:**
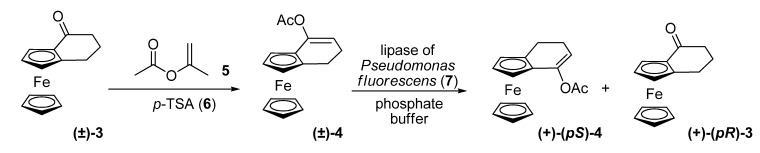
Kinetic resolution of (±)-[4](1,2)ferrocenophan-1-one (**3**).

In a related substance, racemic acetate **8**, *Porcine pancreatic* lipase, *Candida cylindracea* lipase and lipase **7** did not give satisfactory results. Lipase-MY (*Candida cylindracea*, **9**) in diisopropyl ether, on the other hand, allowed hydrolysis of substrate **(±)-8** to (+)-(1*R*)-1-hydroxy-(*R*)-[4](1,2)-ferrocenophane **(+)-(1*R,pR*)-10** with 99% ee at 19% yield, and recovery of (-)-(1*S*)-1-acetoxy-(*S*)-[4](1,2)ferrocenophane **(-)-(1*S,pS*)-8**, with 30% ee at 40% yield, which represents a selectivity value of 400.

**Scheme 3:**
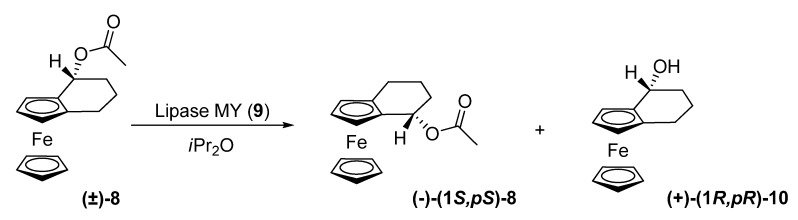
Kinetic resolution of (±)-1-acetoxy-[4](1,2)ferrocenophane (**8**).

On the other hand, the lipase-catalyzed transesterification of **(+)-10** was performed, affording **(+)-(1*R,pR*)-8** with vinyl acetate (or the related product with vinyl butyrate) and **(-)-(1*S,pS*)-10**, obtaining selectivity values up to 430 when lipase PS (**7**) and vinyl butyrate were used. The order of the effect of the acyl donors on the enantioselectivity was found to be vinyl butyrate > vinyl acetate (the rate of esterification was about 2-10 times slower than that using vinyl butyrate). The addition of molecular sieves had a dramatic effect on the reaction rate, by adsorbing the acetaldehyde that is released in the reaction.

Nicolosi and co-workers prepared enantiopure ferrocenyl sulfides possessing planar chirality by means of lipase-assisted resolutions in 1996-1997 [22,23]. This was of special importance given that the few enantiopure sulfur-containing ferrocenes reported in the literature had been mainly prepared by modifying substituents in enantiopure ferrocenes or by diastereoselective synthesis. Nicolosi’s group resolved racemic ferrocenyl sulfides **11** with lipases from *Candida antartica* (Novozym® 435, **13**) or *Mucor miehei* (Lipozyme® IM, **14**) and vinyl acetate (**15**) or vinyl propionate (**16**), with selectivity values up to 34. In the case of phenyl thioether, the reaction with vinyl acetate gave unsatisfactory enantioselectivities, for this reason, a vinyl ester with a bulkier acyl group, namely vinyl propionate (**16**), was chosen, with a significant increase in enantioselectivity when Novozym® 435 (**13**) was used. On the other hand, for the *tert*-butyl thioether, Lipozyme **14** gave better results in terms of reaction rate and enantioselectivities.

**Scheme 4:**
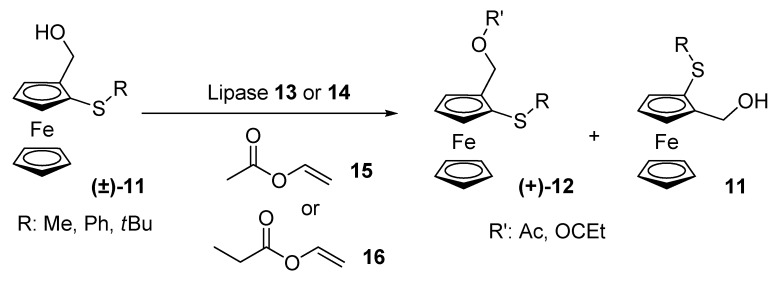
Kinetic resolution of ferrocenyl sulfides **(±)-11**.

With ferrocenyl sulfides **11** in hand (all of them with *S* planar chirality and high ee), the corresponding ferrocenyl sulfoxides were obtained by oxidation with sodium metaperiodate. These sulfoxides were obtained as mixture of diastereomers from methyl and phenyl thioethers, while *tert*-butyl thioether yielded a single product (1*S,pS*-isomer).

Although lipases from different sources had been found to catalyze the kinetic resolution of various *ortho*-substituted hydroxymethylferrocenes, when the substituent is a halogen atom, low enantiomeric excesses had been obtained. The first efficient resolution of such structures appeared in 1998 [24]. Nicolosi and co-workers used a lipase from Candida Antarctica (Novozym® 435, **13**) to resolve 2-hydroxymethyl-1-iodoferrocene **(±)-17**, which is the same lipase successfully employed in the above mentioned resolution of ferrocenyl sulfides.

**Scheme 5:**
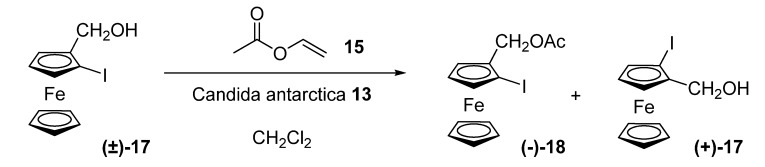
Kinetic resolution of 2-hydroxymethyl-1-iodoferrocene **(±)-17**.

The iodoferrocene **17** was subjected to transesterification with vinyl acetate **15** in dichloromethane in the presence of a lipase from *Candida antarctica* (**13**), which shows *2S*-stereopreference. The resolution reached satisfying levels for preparative uses (E = 67)., and the reaction could be carried out in a multigram scale until substrate conversion of 52%, which afforded unreacted alcohol **(+)-17** with 96% ee and ester **(-)-18** with 89% ee. The enantiomers of 2-hydroxymethyl-1-iodoferrocene could be subjected to copper-assisted substitution reactions to give new enantiopure 1,2-disubstituted ferrocenes, as well as to coupling to afford 2,2’-disubstituted-1,1’-biferrocenes.

Studies on the influence of additives on the outcome of enzymatic reactions are not frequent in the literature. Until 2008, few efforts have been made in order to improve an enzymatic kinetic resolution of a ferrocene-substrate with planar chirality. In that year, Aribi-Zouioueche, Riant and co-workers studied the effects of various additives on the transesterification enzymatic resolution of **(±)-11** using *Candida rugosa* lipase as a catalyst [25].

**Scheme 6:**
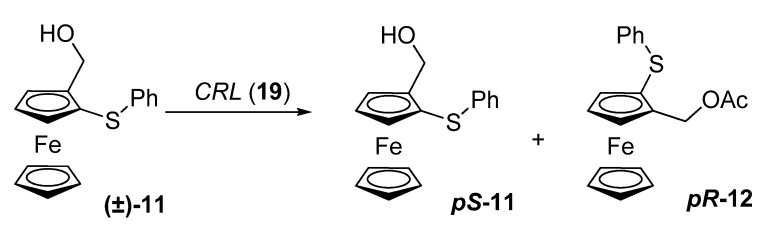
Kinetic resolution of ferrocenyl sulfides **(±)-11**.

The first thing they noticed was that molecular sieves had beneficial effects on the reaction rate and a slight improvement in the selectivity factor, while the addition of water or ethylene glycol completely inhibited the reaction, concluding that the quantity of residual water in the reaction media had a strong influence on both the activity and the selectivity of the process.

Then, they examined the effect of the addition of commercially available *O*-(4-chlorobenzoyl)hydroquinine (**20**), which meant a large enhancement of the reactivity and selectivity of the lipase: an *E*-value of 143 could be reached at 53% conversion with vinyl acetate as the acylating agent in toluene. In *tert*-butyl methyl ether, cinchonidine (**21**) gave the best selectivity. With isopropenyl acetate as acylating agent, on the other hand, the best selectivities were reached with quinine (**22**) and cinchonine (**23**) in toluene. No reaction took place when the experiments were carried out in the presence of an additive, an acylating agent and without any addition of the enzyme. Thus, the interaction of the additive and the lipase was responsible for both reactivity and enantioselectivity of the catalytic system. It was noticed that the rationalization of the impact of the structure of the alkaloid derivatives on the selectivity of the reaction constituted a severe difficulty.

**Figure 1:**
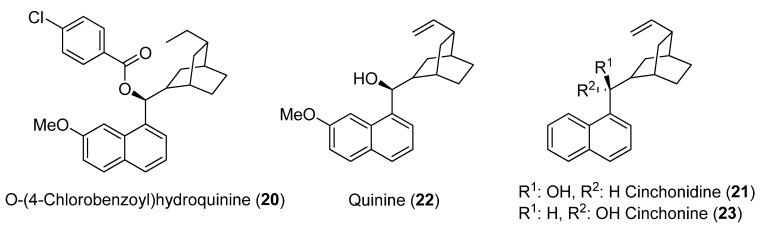
Additives studied in the kinetic resolution of 2-hydroxymethyl-1-phenylthioferrocene (**11**).

Simple organic bases were also selected as additives in order to check the influence of a simple Lewis base on the reactivity of the system. Triethylamine and pyridine gave very little differences with the reference reactions. Stronger Lewis bases such as DMAP completely inhibited the reaction. DABCO significantly increased the enantioselectivity; it could be postulated that this fragment of the alkaloid might intervene in the catalytic cycle to facilitate the access of the substrate to the active site of the enzyme.

In 2009, the same group further optimized the kinetic resolution of 2-hydroxymethyl ferrocenyl sulfides **(±)-11** (see Scheme 4, Scheme 6) in order to offer a simple and scalable protocol for the production of both enantiomers. This kinetic resolution had been studied by Nicolosi *et al*., albeit with modest selectivities (*E* < 40) [26].

The *Candida cylindracea* lipase **24** gave an excellent selectivity of *E* = 267, albeit with very low reactivity. Immobilized *Candida antarctica* lipase (**13**, 40 mg per mmol of substrate) allowed to reach a 48% conversion of the starting alcohol after 72 h and a selectivity of *E* = 152. This result represents a significant improvement over previously published data. It was also found that a high concentration of the acylating agent is susceptible to induce the reversibility of the enzymatic reaction; therefore, a lesser amount of vinyl acetate with an increased amount of the lipase effected an increase in both conversion and selectivity. Screening of the acylating agent and solvent allowed them to conclude that isopropenyl acetate in TBME gave the best results, reaching an optimized conversion after 24 hours with a selectivity value of 168.

When tests were conducted in order to find out the feasibility of recovering the lipase, they concluded that, even if recovered by filtration, lipase always showed high selectivities, but a decrease of reactivity was always observed, thus preventing its effective reuse in successive acylation reactions.

Both enantiomers of sulfide **11** were oxidized to the corresponding sulfoxides **25** with an equimolecular amount of *m*-CPBA (**26**), giving only one diastereomer in each case. The obtained sulfoxides **25** were then used in a trans-metallation-electrophilic sequence to prepare the 2-substituted ferrocenyl alcohols **27**.

**Scheme 7:**
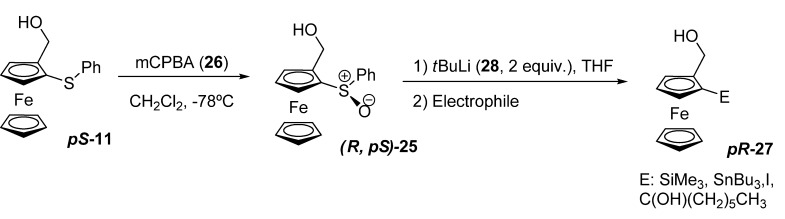
Synthesis of 1,2-disubstituted ferrocenes with planar chirality **27**.

## 3. Non-Enzimatic Kinetic Resolutions

The first non-enzymatic kinetic resolution of a ferrocene with planar chirality was reported, to the best of our knowledge, in 2003, when Uemura and co-workers prepared *C_2_*-symmetric cyclic selenides **29** and **30** having an optically active binaphthyl skeleton and converted them into the corresponding selenoxides **31** and **32**, by sequential use of selenium (**35**) and lithium triethylborohydride (**36**), and *meta*-chloroperbenzoic acid (**26**) and potassium carbonate (**37**) [27].

With these selenoxides in hand, they firstly investigated the kinetic resolution of 2-oxazolin-2-ylferrocenylphosphine **38** using the selenoxide **31**. Reactions were carried out in CCl_4_ at rt for 24 h. the addition of phenol slightly improved the process, with a selectivity factor of 2.3. When selenoxide **32** was used, no asymmetric induction occurred.

**Scheme 8:**
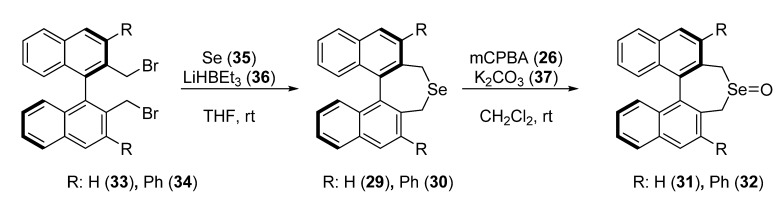
Synthesis of cyclic selenoxides **31** and **32**.

**Scheme 9:**
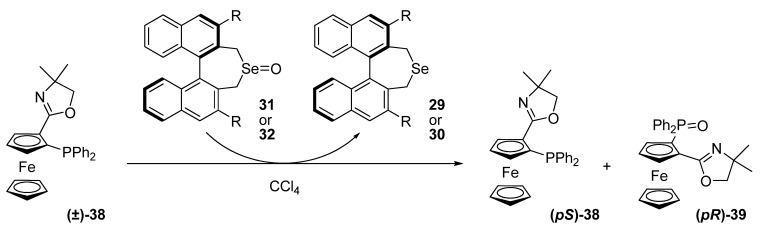
Kinetic resolution of 2-oxazolin-2-ylferrocenylphosphine (**38**).

Other racemic oxazolinylferrocenylphosphines and *N,N*-dimethylaminomethylferrocenylphosphine were investigated for this kinetic resolution using **31** or **32**. Even with the addition of phenol, worse results than with substrate **38** were obtained.

The first example of a highly enantioselective metal-catalyzed kinetic resolution of planar-chiral ferrocene substrates had to wait until 2006. Moyano and co-workers published a kinetic resolution of 2-substituted 1-vinylferrocenes **40** *via* asymmetric dihydroxylation (AD) [28]. Theoretical calculations on the AD of styrene show that in the transition state the phenyl and the alkene moieties present a coplanar geometry. They reasoned that a similar coplanarity requirement should be operative in the AD of 2-substituted vinyl ferrocenes. If this was the case, the presence of a bulky substituent adjacent to the vinyl group would strongly destabilize one of the two limiting planar conformations of the molecule, so that for a given enantiomer of a 2-substituted-1-vinylferrocene one face of the olefin would be much more reactive than the other.

**Scheme 10:**
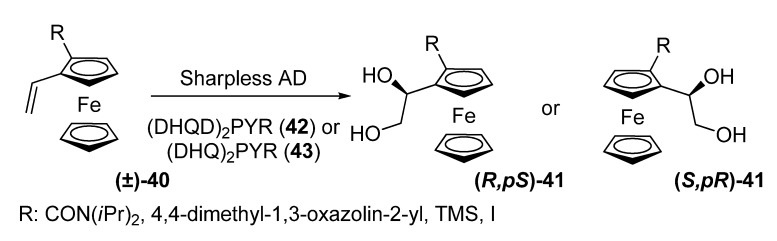
Kinetic resolution of 2-substituted 1-vinylferrocenes **40** *via* AD.

Indeed, the resolution of compounds **40** took place with good enantioselectivity factors (up to 62,3). Clear-cut kinetic resolutions were achieved in all instances, although the *E* values were dependent both on the nature of the 2-substituents and on the AD ligand. The (DHQD)_2_PYR ligand **42** was more selective for a given olefin than the (DHQ)_2_PYR **43**. The enantioselectivity factor of the kinetic resolution was clearly affected by the bulkiness of the 2-substituent, ferrocenylethenes with bulkier substituents (CON(*i*Pr)_2_, 4,4-dimethyl-1,3-oxazolin-2-yl) showing higher enantioselectivity factors than the less bulky ones (TMS, I).

The same year, Ogasawara *et al*. reported the kinetic resolution of planar-chiral 1,1’-diallylferrocene derivatives **44** by Mo-catalyzed asymmetric ring-closing metathesis (ARCM) [29,30].

The ferrocene substrates chosen for this study possess a trisubstituted *η*^5^-(C_5_H_2_-1-allyl-2,4-R^1^_2_) ligand, which constructs a planar-chiral environment in the ferrocenes, and a monosubstituted *η*^5^-cyclopentadienyl ligand with an allylic side chain. A readily available chiral molybdenum species (**45**) was chosen as an asymmetric metathesis catalyst.

**Scheme 11:**
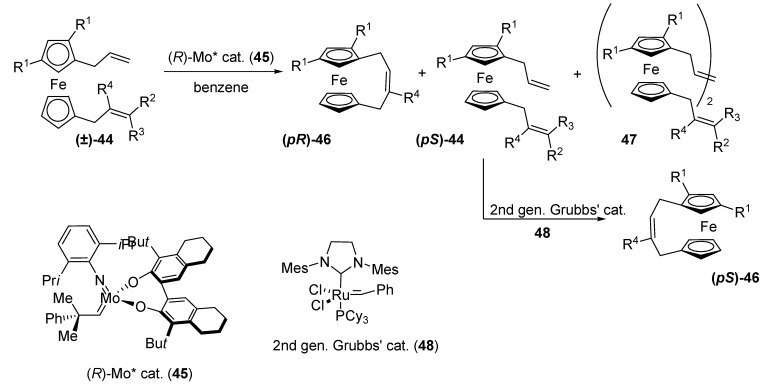
ARCM kinetic resolution of planar-chiral ferrocenes **44** and metathesis catalysts **45** and **48**.

Enantioselectivity in the ARCM kinetic resolution was strongly dependent on the structure of the allylic group in the monosubstituted cyclopentadienyl moiety. When this group was not substituted (R^2^=R^3^=R^4^=H, **44a**), the selectivity of the reaction was very low. The RCM reaction of **44b**, which had a crotyl group, showed slightly better enantioselectivity. With a cinnamyl group (**44c**), enantioselectivity was further improved, although diluted conditions were required to suppress formation of metathesized homodimer **47**. *E* was estimated to be 6.4.

The enantioselectivity was dramatically improved by introducing a methallyl moiety (R^2^=R^3^=H, R^4^=Me, **44d**). The bridged ferrocene **46d** was obtained in nearly enantiomerically pure form (>99.5% ee) in 23% yield, which represented a selectivity value of >500. However, diluted conditions were needed in order to minimize the dimer **47** formation. Somewhat higher temperature (50 ºC) was required to gain a reasonable reaction time, being the enantioselectivity still excellent (*E* = 183). Unreacted enantioenriched **44** could be transformed into **46** with the second-generation Grubbs catalyst.

In 2009, the first organocatalytic kinetic resolution of a planar-chiral ferrocenecarbaldehyde was reported. Rios, Moyano and co-workers described the resolution of 2-(2-pyrimidyl)-ferrocenecarbaldehyde **49** [31]. When the racemic aldehyde **49** was reacted with acetone in DMSO as a solvent and L-proline (**52**) as a catalyst, they obtained enantioenriched starting material, together with two different optically active reaction products: the crotonized adduct **50** and aldol **51**, which was obtained in a highly diastereopure fashion. They found (*pR*)-stereochemistry for the recovered starting material and an (*R,pS*) conﬁguration for the aldol.

**Scheme 12:**
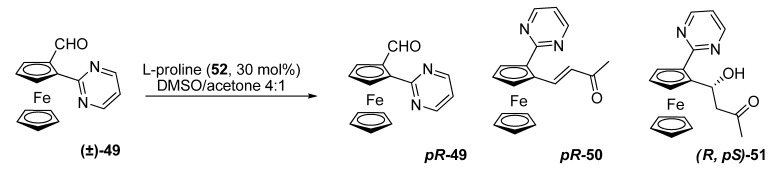
Organocatalytic kinetic resolution of 2-(2-pyrimidyl)ferrocenecarbaldehyde (**49**).

The selectivity factor for this resolution was *E* = 9.2. The kinetic resolution took place, therefore, with moderate selectivity. The observed stereochemical outcome of the resolution ﬁt reasonably well within the mechanistic model commonly accepted for proline-catalyzed aldol reactions.

## 4. Conclusions

In summary, we have described a whole set of methodologies for the synthesis of planar chiral ferrocenes *via* kinetic resolution. These resolutions can be made *via* enzymatic resolution in a classical way, or *via* chemical kinetic resolution. In both cases, the final planar chiral ferrocenes can be obtained with excellent selectivities. In any case, this field remains open, as the importance of planar chiral ferrocenes in chemistry as catalysts and/or building blocks is increasing. This fact has encouraged different groups to focus their research efforts in the development of new and improved methodologies.
